# Comparative study of extension area based methods for spectrophotometric determination of desmopressin acetate in the presence of its acid-induced degradation products

**DOI:** 10.1186/s13065-022-00906-x

**Published:** 2022-12-18

**Authors:** Khadiga M. Kelani, Ahmed M. Wafaa Nassar, Gamal A. Omran, Samir Morshedy, Wael Talaat

**Affiliations:** 1 Analytical Chemistry Department, Faculty of Pharmacy, Cairo University, Kasr El-Aini St., Cairo, P.O. Box 11562 Egypt; 2 Pharmaceutical Analytical Chemistry Department, Faculty of Pharmacy, Modern University for Technology and Information (MTI), Cairo, Egypt; 3 Pharmaceutical Analytical Chemistry Department, Faculty of Pharmacy, Damanhour University, Damanhour, Egypt

**Keywords:** Desmopressin acetate (DPA), First derivative, Derivative ratio, Ratio difference, Mean centering, Dual wavelength

## Abstract

Desmopressin acetate (DPA) is a synthetic analogue of vasopressin used in the treatment of diabetes insipidus, bedwetting, hemophilia A, and elevated levels of urea in the blood. Sensitive and selective stability-indicating methods are needed to be developed and validated for its assay pure and pharmaceutical dosage forms in the presence of its degradation products as no method has been reported for its determination in the presence of its degradants. This work describes a comparative study of five simple stability-indicating spectrophotometric techniques for determination of DPA in presence of its acid-degradation products (acid-degradants) without prior separation. The proposed spectrophotometric techniques (First derivative, Derivative ratio, Ratio difference, Mean centering and Dual wavelength) were developed and validated according to ICH guidelines. Acid degradation was carried out with 0.1 N HCl; the acid-degradants were separated on TLC plates and the acidic degradation pathway was established by IR, H-NMR and MS techniques. The TLC method was based on the separation of DPA and its acid-induced degradation products on silica gel plates using methanol: water (80:20, v/v) as a developing system and UV detection at 254 nm. All assay suggested methods were successfully applied for quantitation of DPA in pure and tablet forms. They are specific, sensitive, precise and accurate. They showed good linearity in the concentration range of 1–14 µg/mL with good correlation coefficients, and limit of detection (LOD) of 0.304, 0.274, 0.167, 0.248 and 0.199 and limit of quantitation (LOQ) of 0.920, 0.829, 0.506, 0.751 and 0.604) for each method, respectively. These methods were successfully applied for the simultaneous determination of DPA in its pure and tablet dosage form in the presence of its acid-degradants. The results obtained were statistically comparable with those of reported HPLC assay method; no significant differences were observed with relevance to accuracy and precision. All the methods are sensitive, selective and can be used for the routine analysis of DPA in its pure and dosage forms.

**Supplementary Information:**

The online version contains supplementary material available at 10.1186/s13065-022-00906-x.

## Introduction

**Fig. 1:**
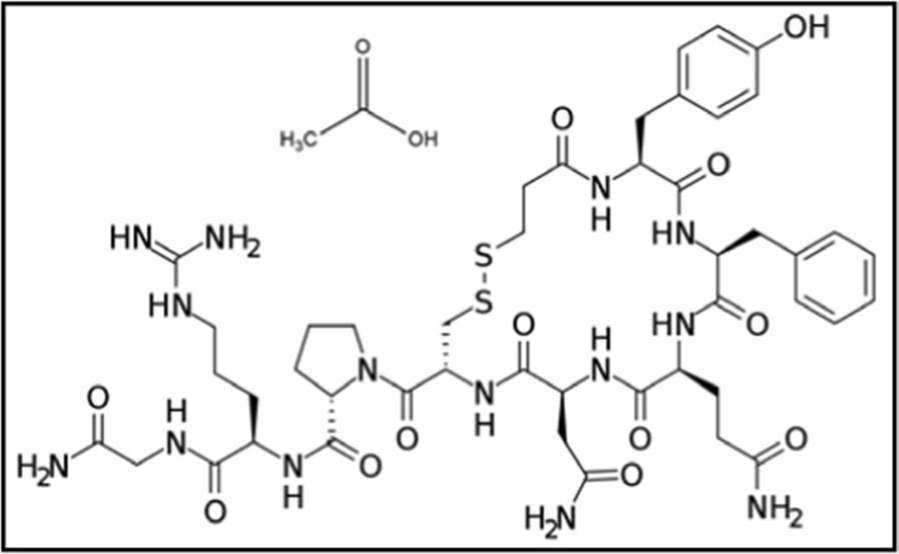
Structural formula of Desmopressin acetate (DPA). Molecular formula: (C_46_H_64_N_14_O_12_S_2_, C_2_H_4_O_2_); molecular weight: 1129.3

Desmopressin acetate (DPA) is {acetic acid-(2S)-N-[(2R)-1-[(2-amino-2-oxoethyl) amino]-5-(diaminomethylideneamino)-1-oxopentan-2-yl]-1[(4R,7S,10S,13S,16S)-7-(2-amino-2-oxoethyl)-10-(3-amino-3-oxopropyl)-13-benzyl-16-[(4-hydroxyphenyl)methyl]-6,9,12,15,18-pentaoxo-1,2-dithia 5,8,11,14,17-pentaza-cycloicosane-4-carbonyl] pyrrolidine-2-carboxamide} [1] (Fig. 1). This drug is used in treatment of diabetes insipidus, bedwetting, hemophilia A, and elevated levels of urea in the blood [2].

It is administered by parenteral, oral and nasal routes in various doses: 20–40 µg (parenteral), 100–200 µg (oral), and 20–40 µg (nasal). which shows limited oral (< 1%) and nasal (< 3.4%) bioavailabilty. Its reduced bioavailability by oral and nasal routes is attributed to enzymatic degradation by gut lumen and nose mucosal tissues enzymes as well as to its low lipophilicity [3, 4]. This enzymatic degradation leads to substantial pre-systemic drug breakdown after oral administration [5].

Many methods were reported for quantitative determination of DPA alone or in combination with others; including HPLC [6–16], spectrophotometric [17], electrochemical [18–23] methods and spectroflourometric method [24]. However, it is sensitive to acid degradation [7] and hence selective stability-indicating methods are needed to be developed and validated [25–28]. Stress testing is required to be undertaken by the International Conference on Harmonization (ICH) guideline entitled “Stability Testing of New Drug Substances and Products” to establish the stability characteristics of the active substance [26, 27]. It suggests that degradants formed under different conditions should be identified and degradation pathways elucidated. Therefore, the objective of this work was to develop simple stability-indicating methods for the determination of DPA in pure and pharmaceutical dosage forms in presence of its acid-degradants; to the best of our knowledge—no method has been reported for the determination of DPA in the presence of its degradants. In this study different spectrophotometric stability indicating assay methods were developed for determination of (DPA) in pure and pharmaceutical dosage form in presence of acid-degradants. Pathway of DPA acid-degradation was established by IR, NMR and mass spectroscopy. All the suggested methods were validated as per ICH guidelines [26, 27].

## Experimental

### Instruments

Double beam UV–Visible spectrophotometer (Shimadzu 1650, Japan) connected to IBM compatible computer, software UV-Probe Ver. 2.1, MATLAB® version R2013b and PLS-Toolbox; hot plate (Torrey Pines Scientific, USA), Rota-Vapor SCI-Logics (RE-100-PRO) with Buchi pump; Aluminum TLC plates precoated with silica gel 60 GF254 (20 × 20 cm), (Merck, Darmstadt, Germany) with its chromatographic tank (25 × 25 × 9 cm); and Jenway, 3510 pH meter (Jenway, USA).

### Materials and chemicals

Pure DPA (99.30%) was kindly provided by Sigma Pharmaceutical Industrial Company, Cairo, Egypt. Omegapress® tablets labeled to contain 0.1 mg of DPA per tablet (batch number 33019) manufactured by Sigma Pharmaceutical Industrial Company was purchased from local market. Hydrochloric acid, sodium hydroxide and methanol were the products of from El-Nasr Pharmaceutical Co., Cairo, Egypt.

### Standard solutions

Stock solution of (100 µg/mL) for DPA was prepared by dissolving 10 mg of DPA in 100 mL methanol. Different sets of working solution at various concentrations were prepared by appropriate dilution of the stock solution in methanol.

### Procedures

#### Acid-degradation of DPA

Accurately weighed one hundred (100) mg of pure DPA powder was treated with 5 mL 0.1 N HCl in a 100-mL round bottom flask, and the solution was heated at 60 °C under reflux for 6 h. After cooling to room temperature, the solution was adjusted to pH 7 with 0.1 N HCl and evaporated to dryness under vacuum. The obtained residue was extracted three times each with 25 mL methanol, filtered into 100-mL volumetric flask and the volume was completed to the mark with methanol and mixed well to obtain a stock solution containing acid-degradants derived from 1 mg/mL of DPA.

#### Test of complete degradation of DPA

Complete degradation of DPA was checked by TLC using methanol – water (80:20, v/v) as a developing system and UV detection at 254 nm.

#### Analytical profile of acid-degradants

The acid-degradants were characterized using IR, NMR, and mass spectroscopy.

#### Construction of calibration curves (linearity)

Construction of calibration curves followed the methods described by Erk [29]; Choudhari et al. [30]; Hajian, Shams and Kaedi [31]; Afkhami and Bahram [32]; and Fernandes et al. [33], respectively.

##### First derivative method (^1^D) [29]

Aliquots of standard DPA solution in methanol (0.1 mg/mL) equivalent to 0.01–0.14 mg of the drug were added to a series of 10-mL volumetric flasks and diluted to the mark with methanol. First derivative (^1^D) spectra of the drug were recorded against methanol as blank. The amplitude of the trough at 232.6 nm was measured for each concentration, where the acid-degradants read zero absorption. A Calibration curve relating trough amplitude to drug concentration in µg/mL was constructed, and the regression equation was derived.

##### Ratio derivative method (^1^DD) [30]

Aliquots equivalent to 0.01–0.14 mg/mL DPA were accurately transferred from their standard working solutions (100 µg/mL) into a series of 10-mL volumetric flasks then completed to volume with methanol. The spectra of the prepared standard solutions were scanned from 200 to 400 nm and stored in the computer. The stored spectra of DPA were divided by the spectrum of the acid-degradants which is equivalent to 6 µg/mL of DAP. The amplitude of the first derivative trough of (DPA / degradants) was measured at 236 nm. A calibration graph relating the trough amplitude at 236 nm to the corresponding concentrations in µg/mL of DPA was constructed and the regression equation was derived.

##### Ratio difference method (RD) [31]

Aliquots equivalent to 0.01–0.14 mg were accurately transferred from DPA standard stock solution (0.1 mg/mL) into a series of 10-mL volumetric flasks then completed to volume with methanol. The spectra of the prepared standard solutions were scanned from 200 to 400 nm and stored in the computer, for the determination of DPA in presence of its acid-degradation products; the stored spectra of DPA were divided by the spectrum of the acid-degradants (equivalent to 6 µg/mL of DPA). The amplitude difference at 225 and 277 nm (∆P_225–277_) was plotted against the corresponding DPA concentration in µg/mL and the regression equation was computed.

##### Mean centering method (MC) [32]

Aliquots equivalent to 0.01–0.14 mg of DPA working standard solution were accurately transferred into a series of 10-mL volumetric flasks then completed to volume with methanol. The spectra of the prepared standard solutions were scanned from 200 to 400 nm, using methanol as a blank and stored in the computer. The absorption spectra of DPA were divided by the spectrum of the acid-degradants (equivalent to 6 µg/mL of DPA). The amplitude of the mean centered peak of intact/degradant using MATLAB® [17] was measured at 236 nm. A calibration graph relating the peak amplitude to the corresponding concentrations in (µg/mL) of DPA was constructed and the regression equation was computed.

##### Dual wavelength method (DW) [33]

Aliquots of standard DPA solution in methanol (0.1 mg/mL) containing 0.01 – 0.14 mg of the drug were added to a series of 10-mL volumetric flasks and then diluted to the mark with methanol. In zero order spectra, the difference absorbance at 237 and 273 nm was found to be zero for acid-degradants: calibration graphs relating difference absorbance at 237 and 273 nm to the corresponding concentration of DPA were constructed, and the corresponding equation was computed.

## Application to laboratory prepared mixtures

Accurate aliquots of DPA and its acid-degradants solution were transferred from their working solutions into a series of 10-mL volumetric flasks to prepare mixtures containing different ratios of both. The volumes were completed with methanol. The spectra of the prepared series from 200 to 400 nm were recorded and stored. The stored spectra were divided by the divisor as before. The concentrations of DPA were calculated as described under linearity for each proposed method.

### Application to tablet dosage form

Ten Omegapress ® 1 mg tablets were accurately weighed and finely powdered manually in a mortar. An appropraite amount of the powder equivalent to 10 mg of DPA were transferred to 100-mL flask, shaken three times with 25 mL methanol for 15 min then filtered into 100-mL volumetric flask and the volume was adjusted to the mark with water. Accurately measured 1 mL of the prepared solution was transferred into 10-mL volumetric flask and the volume was completed to the mark with water to obtain a concentration of 0.1 mg/mL. The solution was analyzed using the procedure described under method 1,2,3,4 and 5.

## Results and discussion

### Acid-degradation of DPA and test for complete degradation

In the present work, the acid hydrolysis of DPA by HCl has been studied to investigate its stability characteristics. Complete degradation was achieved by refluxing with 0.1 N HCl at 60 ^°^C for approximately 6 h as verified by the proposed TLC method using methanol: water (80:20, v/v) as a developing system and UV detection at 254 nm.

## Analytical profile of acidic degradation

**Fig. 2:**
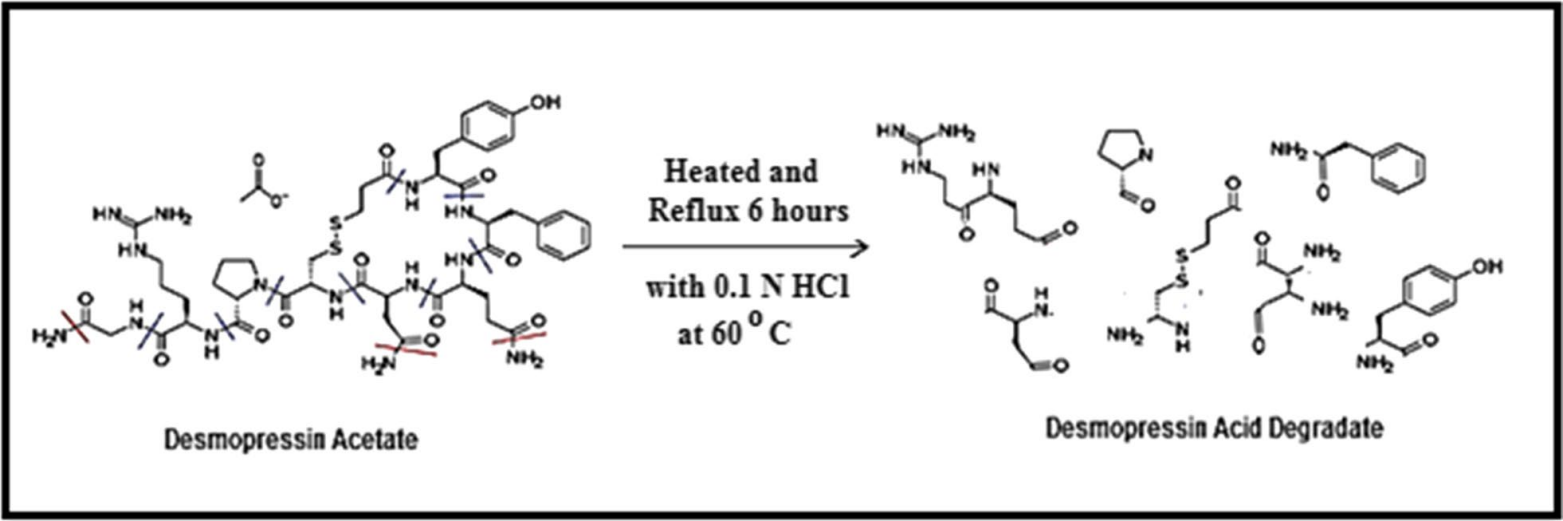
Suggested pathway of acid-degradation of DPA

The structures of the acid-degradants were elucidated by IR, NMR, and MS techniques. A suggested route of acid degradation is shown in (Fig. 2).

### Elucidation of degradation pathway using IR technique

IR spectrum of the intact DPA (Additional file 1: Fig. S1), showed a peak of phenolic (−OH) at 3343.20 cm^−1^ and a peak of (-CN) at 2217.51 cm^−1^. However, IR spectrum of degradants (Additional file 1: Fig. S2), showed disappearance of (C = O) stretch of amide at 1627.35 cm^−1^ and appearance of (C = O) stretch of carboxylic acid at 1779.68 cm^−1^. Also, the IR spectrum of degradants showed appearance of broad band of carboxylic acid (−OH) at 3441.53 cm^−1^ indicating the cleavage of amide linkage with formation of carboxylic group.

### Elucidation of degradation pathway using ^1^H NMR technique

The ^1^H NMR of the intact DPA in dimethyl sulfoxide (DMSO) (Additional file 1: Fig. S2), showed triplet signal of six protons of the two aliphatic (-CH_2_-) groups at 1.236–1.422 ppm, quartet signal of four protons of the two aliphatic (−CH_3_) groups at 3.819–3.982 ppm, singlet signal of one proton of the vinyl group at 7.632 ppm, multiplet signals of two aromatic protons at 7.749–7.927 ppm and 2 signals of two phenolic (−OH) groups at 9.668–10.993 ppm, While ^1^H NMR of intact desmopressin acetate in deuterated dimethyl sulfoxide (DMSO) in (Additional file 1: Fig. S4), showed disappearance of the 2 phenolic (−OH) signals. The ^1^H NMR of degradants in dimethyl sulfoxide (DMSO) (Additional file 1: Fig. S5), showed appearance of (−OH) carboxylic acid signal at 12.188 ppm, while ^1^H NMR of degradants in deuterated dimethyl sulfoxide (DMSO) (Additional file 1: Fig. S6) showed disappearance of the 2 phenolic (−OH) and carboxylic acid (−OH) indicating the cleavage of amide linkage with formation of carboxylic group.

### Elucidation of degradation pathway using mass spectroscopy

Mass Spectroscopy was performed for degradants; molecular ion peaks was obtained at m/z = 1129.3, indicating that their molecular weight is 1129.3 of DPA as shown in (Additional file 1: Fig. S7).

## Methods development and application

Five different spectrophotometric methods were developed and applied for the determination of DPA in presence of its acid-degradation products. These methods are based on those reported by Erk [29]; Choudhari et al. [30]; Hajian, Shams and Kaedi [31]; Afkhami and Bahram [32]; and Fernandes et al. [33], respectively. A comparative study between the methods was carried out to select the most sensitive and applicable method(s). The spectra of the drug at zero order showed high degree of interference with the degradant spectrum which hinders the direct UV determination of DPA in presence of its acid-degradation products. The main propose of application of these methods were to establish a stability indicating assay with high selectivity, precision and sensitivity to determine the drug in its dosage form in presence of its acid-degradation products.

### First derivative method [29]

**Fig. 3:**
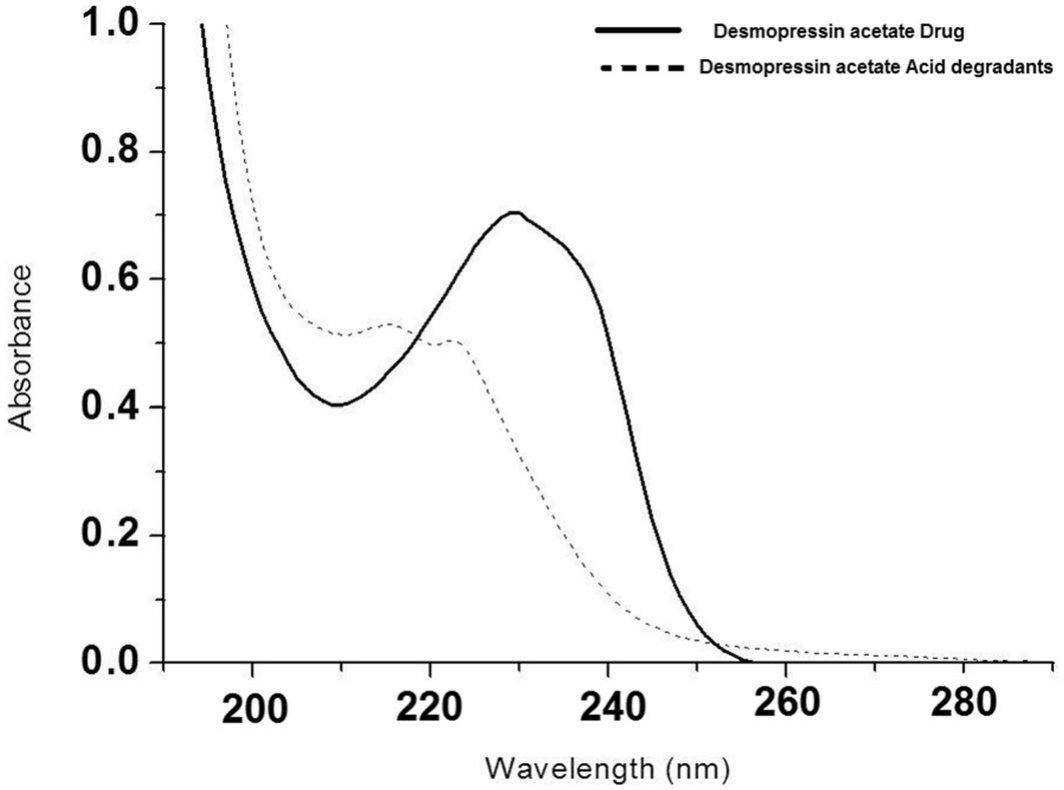
Zero-order absorption spectra of intact DPA. (10 µg/mL) (―) and its acid-degradants (…..) in methanol

**Fig. 4:**
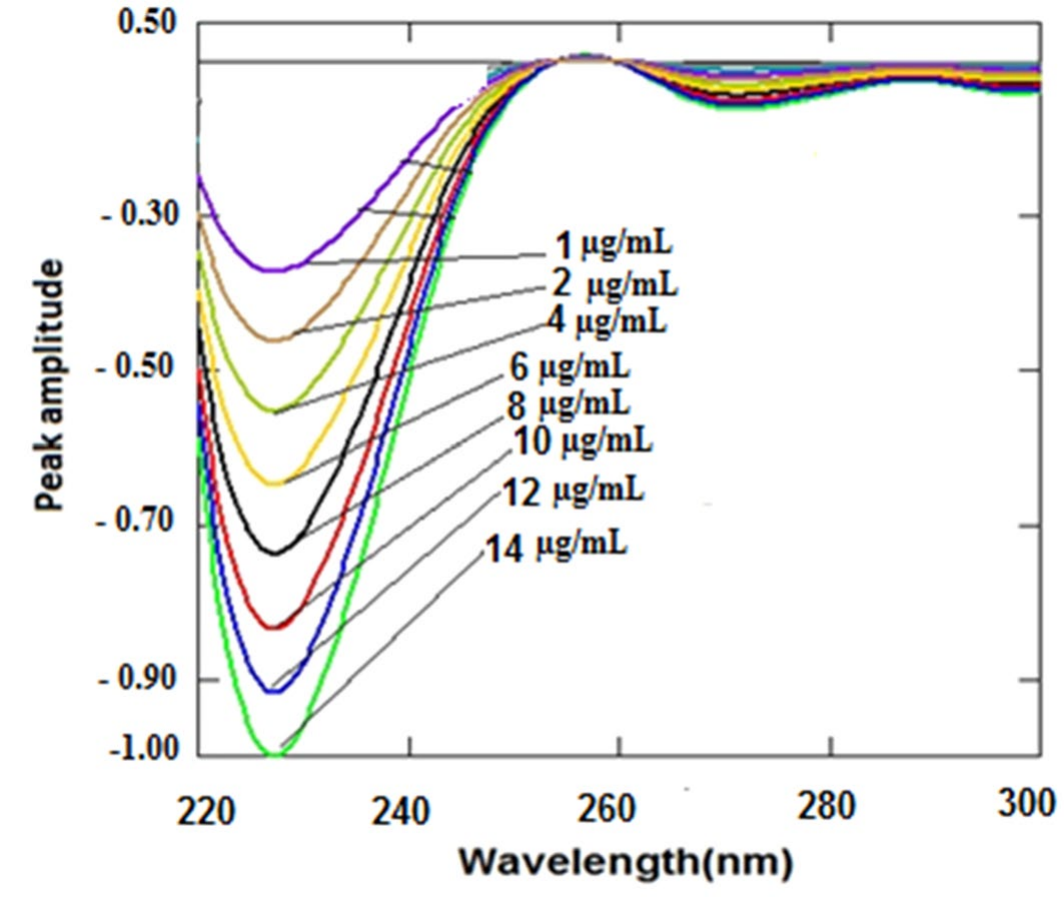
First derivative of the absorption spectra of DPA at various concentrations (1–14 µg/mL)

The zero order absorption spectrum of DPA (10 µg/mL) and its equivalent acid-degradants were recorded against methanol as blank over the range of 200–400 nm, with severe overlapping as shown in (Fig. 3). However, the severe overlapping in zero order spectra can be resolved by conversion of zero-order to first derivative spectra of DPA and its acid-degradants. DPA has a trough at 232.6 nm after smoothed with Δλ = 16 nm and scaling factor = 10 (Additional file 1: Fig. S8) which shows no interference from the degradation products. The linear regression equation for DPA in first derivative graph was:

$$ {\mathbf{y}} \, = \, {\mathbf{0}}.{\mathbf{00163}} \, {\mathbf{x}} \, + \, {\mathbf{0}}.{\mathbf{00064}}\, \left( {{\mathbf{r}} \, = \, {\mathbf{0}}.{\mathbf{9998}}} \right),$$

where y is the peak amplitude values, x is the drug concentration and r is the correlation coefficient. Linearity range, intercept, slope and correlation coefficient for the calibration data were shown in (Fig. 4). The yielded statistical results are summarized in Table 1. This method can determine the drug in presence of up to 70% of the acid-degradants as shown in Table 2.

**Table 1:** Linearity and regression parameters of the proposed methods

Parameters	First derivative	Ratio derivative	Ratio difference	Mean centering	Dual wavelength
Wavelength	232.6 nm	236 nm	225–277 nm	236 nm	237–273 nm
Calibration range	(1–14 µg /mL)	(1–14 µg /mL)	(1–14 µg /mL)	(1–14 µg /mL)	(1–14 µg /mL)
Slope	0.00163	0.0374	0.7191	0.6482	0.0298
Intercept	0.00064	0.0122	0.0652	0.1753	0.0032
Correlation coefficient	0.9998	0.9997	0.9997	0.9998	0.9997
LOD	0.304	0.274	0.167	0.248	0.199
LOQ	0.920	0.829	0.506	0.751	0.604

**Table 2:** Determination of intact desmopressin acetate (DPA) in laboratory prepared mixtures with its degradants by the proposed methods

Conc. of DPA (µg/mL)	Conc. of degradants ( g/mL)	% of degradants	Recovery % of Intact DPA				
			First derivative	Ratio derivative	Ratio difference	Mean centering	Dual wavelength
13	1	7.14	101.58	98.39	101.93	100.80	99.02
12	2	14.28	98.77	101.28	99.04	101.03	100.11
10	4	28.57	99.82	99.92	101.47	100.91	99.23
8	6	42.86	100.39	99.40	100.19	99.92	98.01
6	8	57.14	99.01	100.64	98.62	99.73	101.16
4	10	71.43	98.16	108.59 ^a^	99.95	100.69	98.14
2	12	85.71	95.23 ^a^	114.28 ^a^	101.82	106.24 ^a^	104.97 ^a^
Mean			99.62	99.93	100.43	100.51	99.28
RSD%			1.241	1.117	1.334	0.546	1.209

^a^Under-lined values are out of accepted range and not considered in the calculation of Mean or SD

### Ratio derivative method [30]

**Fig. 5:**
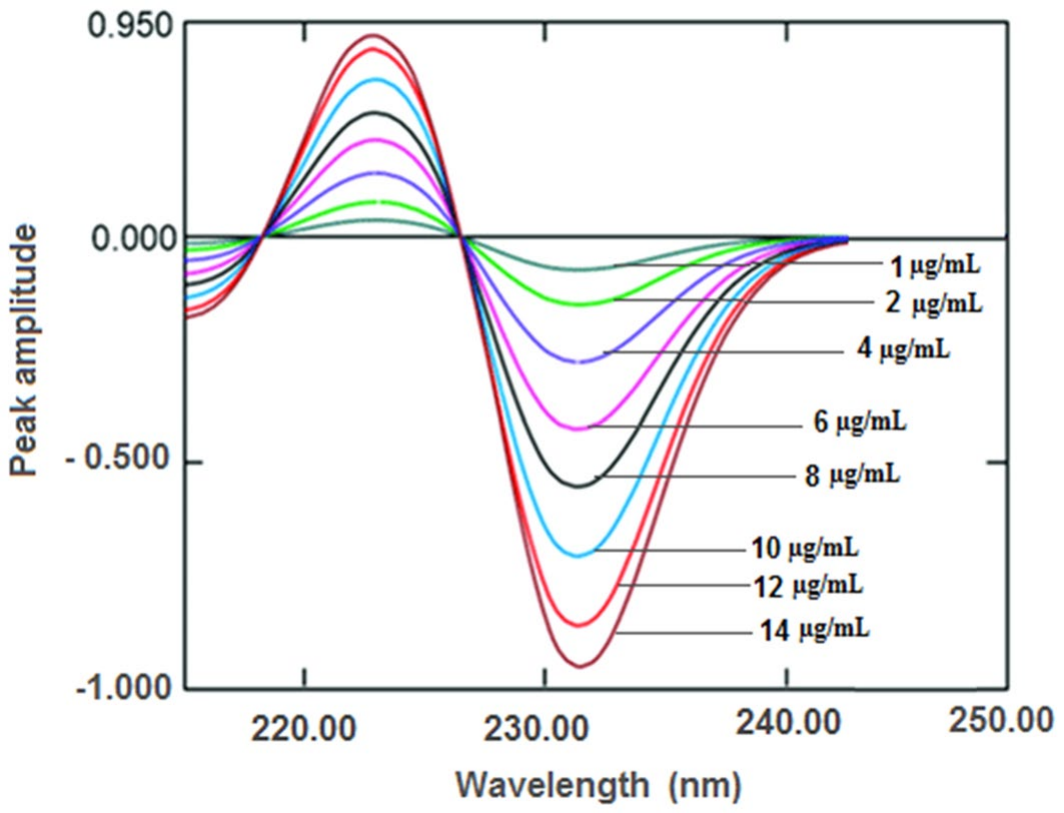
First derivative of ratio spectra of DPA (1–14 µg/mL) using acid-degradants (equivalent to 6 µg/mL of DPA) as divisor and methanol as blank

The method is based on the derivation of the ratio-spectra as shown in (Additional file 1: Fig. S9) to resolve the interference. Different concentrations of DPA and different divisor concentrations of degradates were tried. Careful choice of the divisor is mandatory and the selected divisors should compromise between minimal noise and maximum sensitivity. The divisor concentration (equivalent to 6 µg/mL of DAP) gave the best results as shown in (Fig. 5). The main advantage of the ratio-spectra derivative spectrophotometry is the chance of doing simple measurements in correspondence of peaks so it permits the use of the wavelength of highest value of analytical signals (a maximum or a minimum). The calibration graph for the method was constructed by plotting peak amplitude at 236 nm against the corresponding concentration of DPA. The linear regression equation for DPA in ratio derivative graph was:

$$ {\mathbf{y}} \, = \, {\mathbf{0}}.{\mathbf{0374}} \, {\mathbf{x}} \, + \, {\mathbf{0}}.{\mathbf{0122}} \,\left( {{\mathbf{r}} \, = \, {\mathbf{0}}.{\mathbf{9997}}} \right),$$

where y is the peak amplitude values, x is the drug concentration and r is the correlation coefficient. The yielded statistical results are also summarized in Table 1. This method determine the drug in presence of up to 60% of the acid- degradants as shown in Table 2.

### Ratio difference method [31]

The method starts by scanning zero order spectra of the prepared standard solution of DPA and its acid-degradants in methanol. The divisor concentration of acid-degradants equivalent to 6 µg/mL DAP gave the best results. Two wavelengths (225 and 277 nm) were chosen on the ratio spectra**,** difference between these two wavelengths (∆P_225– 277_) was calculated and good linearity was obtained.

The linear regression equation for DPA in ratio difference graph was:

$$ {\mathbf{y}} \, = \, {\mathbf{0}}.{\mathbf{7191}} \, {\mathbf{x}} \, + \, {\mathbf{0}}.{\mathbf{0652}} \,\left( {{\mathbf{r}} \, = \, {\mathbf{0}}.{\mathbf{9997}}} \right),$$

where y is the peak amplitude values, x is the drug concentration and r is the correlation coefficient. The yielded statistical results are summarized in Table 1. This method determine the drug in presence of up to 100% of the acidic degradate with lowest LOD = 0.167 and LOQ = 0.506 as shown in Table 2. The ratio difference technique exceeded the other techniques in terms of LOD and LOQ [25–28] “the smaller values of LOD and LOQ, the more sensitive the methods”, because it eliminates the derivatives step and therefore the signal to noise ratio is enhanced.

### Mean centering method [32]

The ratio spectra were obtained by testing different concentrations of the divisors, but the concentrations (6 μg/mL) of DPA gave minimum noise in ratio spectra and maximum sensitivity. The ratio spectra were mean centered in the range 200–400 nm for DPA (Additional file 1: Fig. S10). The concentration of DPA was calculated by using the regression equation representing the linear relationship between mean centered values at 236 nm, and the corresponding concentrations. The linear regression equation for DPA in mean centering graph was:

$$ {\mathbf{y = 0}}{\mathbf{.6482 x + 0}}{\mathbf{.1753 }}\,\left( {{\mathbf{r = 0}}{\mathbf{.9998}}} \right){\mathbf{,}}$$

where y is the area under peak values, x is the drug concentration and r is the correlation coefficient. The yielded statistical results are summarized in Table 1. This method determines the drug in presence of up to 70% of the acid-degradants as shown in Table 2.

### Dual wavelength method [33]

In this method, determination of intact DPA in presence of its degradation products can be achieved by calculating difference in absorbance in zero order absorption spectra of DPA and its acid-degradants at two selected wavelengths (237 and 273 nm.), when the difference in absorbance at these wave lengths was found to be zero for the degradants, while the intact spectra have the different absorbance values. Determination of DPA at these wavelengths can be achieved without interference to its acid-degradants. Good linearity at (∆P_237-273_) was obtained and the linear regression equation for DPA in dual wavelength graph was:

$$ {\mathbf{y}} \, = \, {\mathbf{0}}.{\mathbf{0298}} \, {\mathbf{x}} \, + \, {\mathbf{0}}.{\mathbf{0032}} \,\left( {{\mathbf{r}} \, = \, {\mathbf{0}}.{\mathbf{9997}}} \right), $$

where y is the difference in the absorption values, x is the drug concentration and r is the correlation coefficient. The yielded statistical results are summarized in Table 1. This method determines the drug in presence of up to 70% of its acid-degradants as shown in Table 2.

## Methods validation

The proposed methods were validated using the ICH guidelines [26, 27]. The validation results are shown in Tables 1, 2, 3 and Additional file 1: Tables S1, S2).

**Table 3:** Statistical comparison between the results obtained by applying the proposed spectrophotometric methods and reported method for determination of DPA

Parameters	First derivative	Ratio derivative	Ratio difference	Mean centering	Dual wavelength	Reported method [6]^***^
Mean	100.21	99.94	100.11	99.78	99.47	98.99
S.D	1.143	0.999	1.036	0.607	1.082	0.409
N*	5	5	5	5	5	5
*t*-test**	2.203 (2.365)	1.931 (2.365)	2.199 (2.365)	2.319 (2.365)	0.907 (2.365)	–
*F*-value**	7.820 (9.117)	5.984 (9.117)	6.426 (9.117)	2.206 (9.117)	7.014 (9.117)	–

*Number of experimental.

**The values in the parenthesis are the corresponding theoretical values of *t* and *F* at (*P* = 0.05).

***Determination of the Content of Desmopressin in Pharmaceutical Preparations by HPLC and Validation of the Method [6].

### Linearity

Calibration curves were constructed using a series of standard solutions in the range 1–14 μg/mL. The linearity’s were achieved and the concentrations of the drug were calculated Table 1.

### Precision

The intraday and interday precision were calculated Additional file 1: Table S1**.**

### Accuracy

The suggested procedures were successfully applied to quantify the drug in a pharmaceutical dosage form Table 1. The validity of the obtained results was assessed by applying the standard addition technique Additional file 1: Table S2.

### Specificity

The proposed methods were capable of determining DPA selectively in its pharmaceutical formulation even in the presence of its acid-degradants Table 2.

### Sensitivity

The proposed methods were capable of determining DPA at the low concentrations of (1 and 14 μg/mL) for the five proposed methods Table 1.

### LOD and LOQ

According to ICH recommendations, the approach based on the SD of the response and slope was used for determining the LOD and LOQ. The experimental values are given in Table 1.

### Statistical analysis

Statistical comparison of the results obtained by the proposed methods and a reported method [6] was shown in Table 3. The calculated t and F values were less than the theoretical ones indicating that there was no significant difference between the proposed and the reported method with respect to accuracy and precision.

## Conclusion

The proposed First derivative, Derivative ratio, Ratio difference, Mean centering and Dual wavelength techniques provided simple, accurate, and reproducible quantitative determination of DPA in pure form and a pharmaceutical formulation (tablets) in the presence of its acid- degradation products and without any interference from excipients. All the methods were found to be sensitive, selective and can be used for the routine analysis of desmopressin acetate (DPA) in their available dosage forms. The methods are also suitable and valid for application in laboratories lacking liquid chromatographic instruments. It was clear that the ratio difference technique has the advantages of being more sensitive compared to the other techniques as it showed smaller LOD and LOQ [25–28] “more small values of LOD and LOQ, more sensitive the methods”; it eliminates the derivatives step and therefore the signal to noise ratio is enhanced. Also, the ratio difference technique showed more specificity and seems to be the simplest one as it does not require special software (MATLAB). Moreover, DPA acid-degradation pathway was established by IR, H-NMR and MS techniques.

## Supplementary Information

**Additional file 1:** Desmopressin acetate in presence of its acid degradants. **Figure S1.** IR spectrum of intact desmopressin acetate (DPA) on KBr disc. **Figure S2.** IR spectrum of desmopressin acetate (DPA) degradants on KBr disc. **Figure S3.** ^1^H NMR spectrum of intact desmopressin acetate in (DMSO). **Figure S4.** ^1^H NMR spectrum of intact desmopressin acetate (DPA) in deuterated (DMSO). **Figure S5.** ^1^H NMR spectrum of desmopressin acetate (DPA) acid-degradants in (DMSO). **Figure S6.** ^1^H NMR spectrum of desmopressin acetate (DPA) Acid-degradants in deuterated (DMSO). **Figure S7.** Mass spectrums of desmopressin acetate (DPA) degradants. F**igure S8.** First-derivative spectra of intact desmopressin acetate (DPA) (—) and its degradation products (--) in methanol. **Figure S9.** First derivative of the absorption spectra of desmopressin acetate (DPA) at various concentrations (1-14 µg/mL). **Figure S10.** Ratio spectra of desmopressin acetate (DPA) (1 -14 µg/mL) using (6 µg/mL) DPA acid-degradants as divisor and methanol as blank. **Figure S11.** Mean centered ratio spectra of desmopressin acetate (DPA) (1–14 µg/mL) using (6 µg/mL) of its degradants as a divisor and methanol as blank. **Table S1.** The intraday and interday precision of the the proposed methods. **Table S2.** Application of standard addition technique to the analysis of Omegapress® tablets using the proposed methods.

## Data Availability

All data generated or analyzed during this study are included in this published article and its supplementary information files listed below.

## Abbreviations

LOD: Limit of detection
LOQ: Limit of quantitation
DPA: Desmopressin acetate
ICH: International Council for Harmonization
^1^DD: Ratio derivative
^1^D: First derivative
RD: Ratio difference
MC: Mean centering
DW: Dual wavelength
