# Supplementary material for: Comparative study of extension area based methods for spectrophotometric determination of desmopressin acetate in the presence of its acid-induced degradation products
Source: BMC Chem. 2022 Dec 18;16(1):117. doi: 10.1186/s13065-022-00906-x (PMC9759903; doi:10.1186/s13065-022-00906-x)
Supplement: Supplementary file 1 — Additional file 1: Desmopressin acetate in presence of its acid degradants. Figure S1. IR spectrum of intact desmopressin acetate (DPA) on KBr disc. Figure S2. IR spectrum of desmopressin acetate (DPA) degradants on KBr disc. Figure S3. 1H NMR spectrum of intact desmopressin acetate in (DMSO). Figure S4. 1H NMR spectrum of intact desmopressin acetate (DPA) in deuterated (DMSO). Figure S5. 1H NMR spectrum of desmopressin acetate (DPA) acid-degradants in (DMSO). Figure S6. 1H NMR spectrum of desmopressin acetate (DPA) Acid-degradants in deuterated (DMSO). Figure S7. Mass spectrums of desmopressin acetate (DPA) degradants. Figure S8. First-derivative spectra of intact desmopressin acetate (DPA) (—) and its degradation products (--) in methanol. Figure S9. First derivative of the absorption spectra of desmopressin acetate (DPA) at various concentrations (1-14 µg/mL). Figure S10. Ratio spectra of desmopressin acetate (DPA) (1 -14 µg/mL) using (6 µg/mL) DPA acid-degradants as divisor and methanol as blank. Figure S11. Mean centered ratio spectra of desmopressin acetate (DPA) (1–14 µg/mL) using (6 µg/mL) of its degradants as a divisor and methanol as blank. Table S1. The intraday and interday precision of the the proposed methods. Table S2. Application of standard addition technique to the analysis of Omegapress® tablets using the proposed methods. [file 13065_2022_906_MOESM1_ESM.docx]

**Additional file 1**

**Additional file of Desmopressin acetate in presence of its acid degradants**


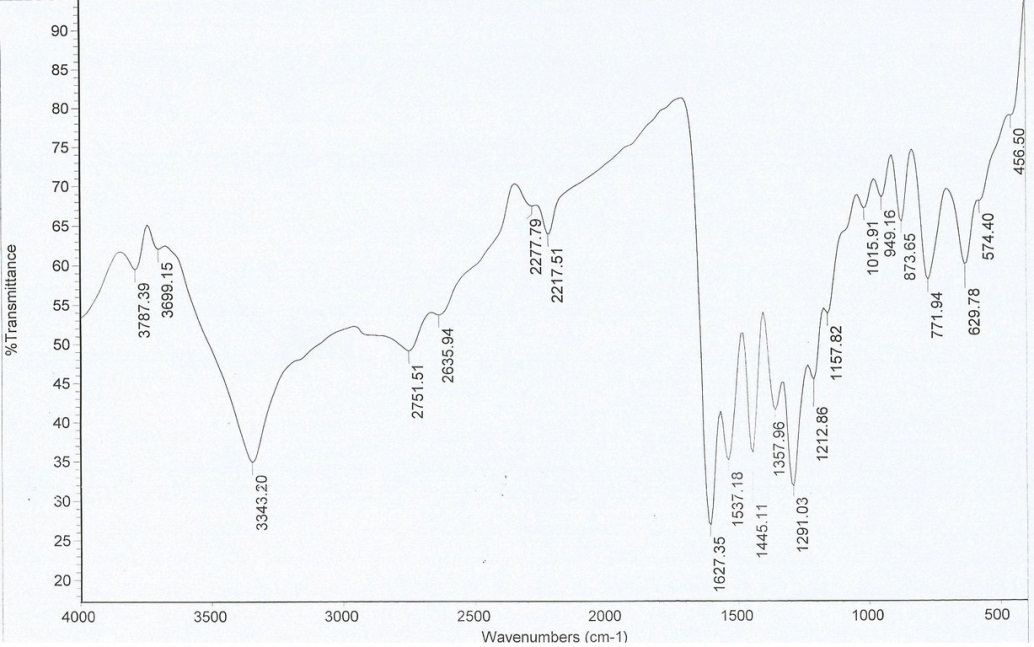


**Figure S1. IR spectrum of intact desmopressin acetate (DPA) on KBr disc.**


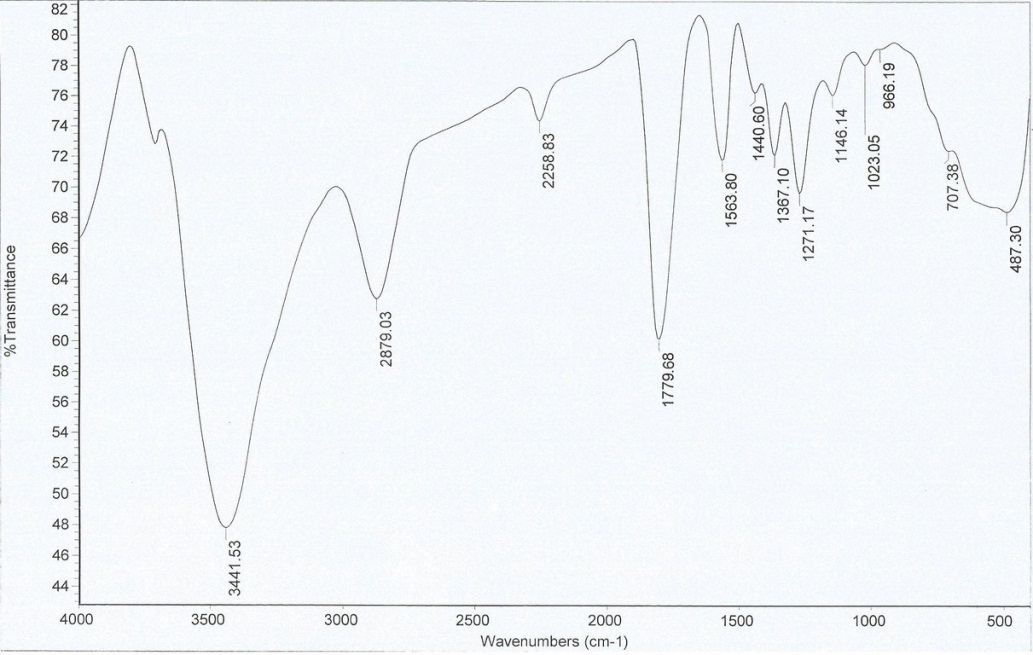


**Figure S2. IR spectrum of desmopressin acetate (DPA) degradants on KBr disc.**


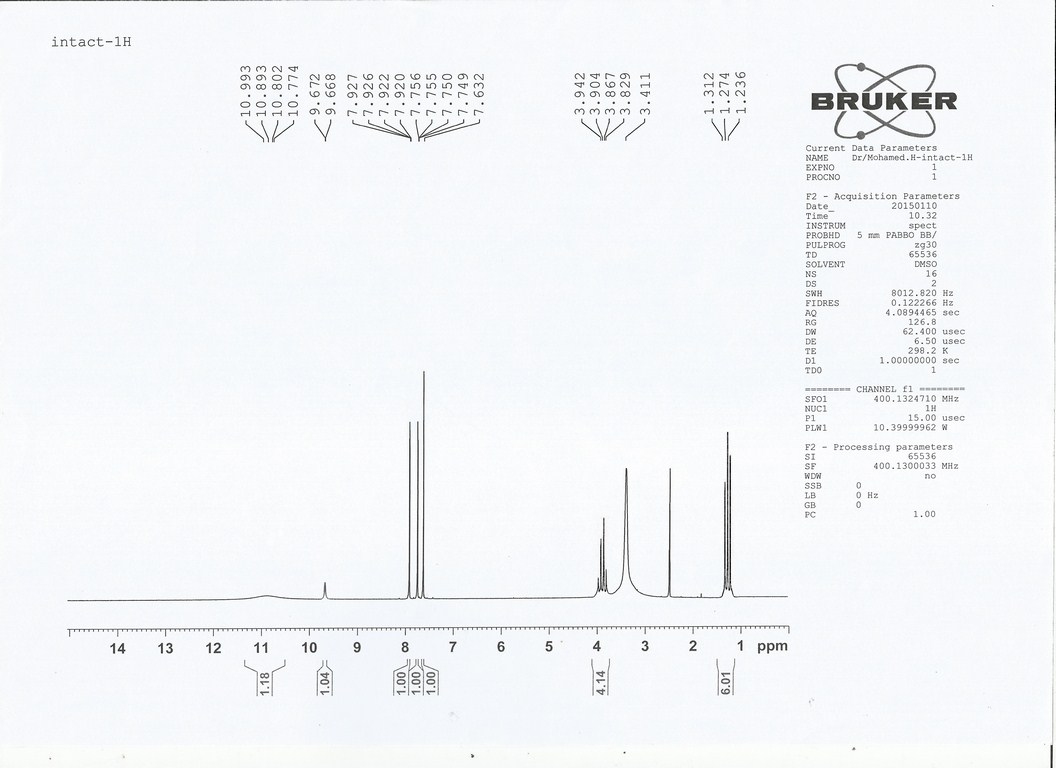


**Figure S3. ^1^H NMR spectrum of intact desmopressin acetate in (DMSO).**


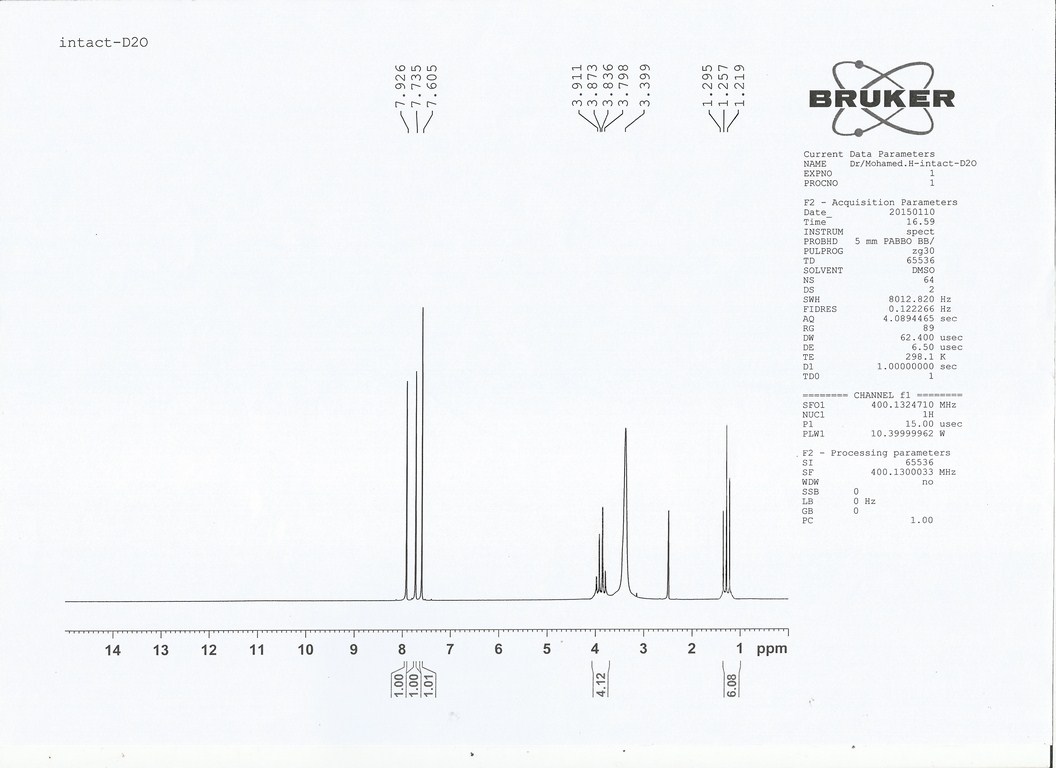


**Figure S4. ^1^H NMR spectrum of intact desmopressin acetate (DPA) in deuterated (DMSO).**


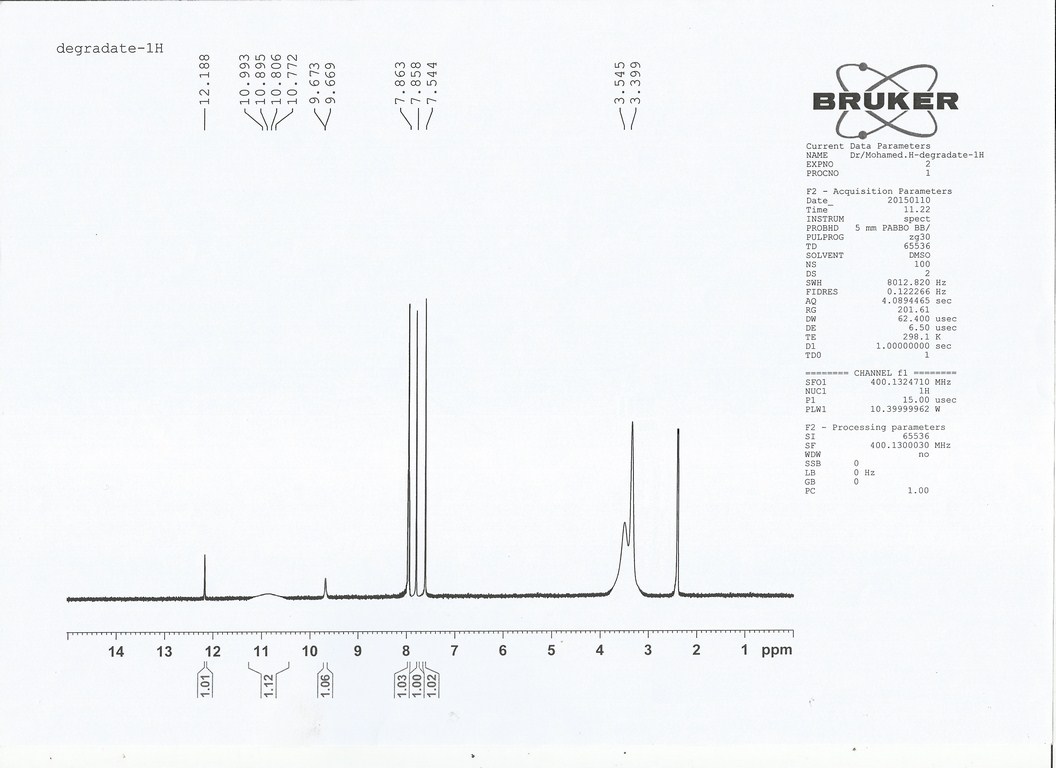


**Figure S5. ^1^H NMR spectrum of desmopressin acetate (DPA) acid-degradants in (DMSO).**

**
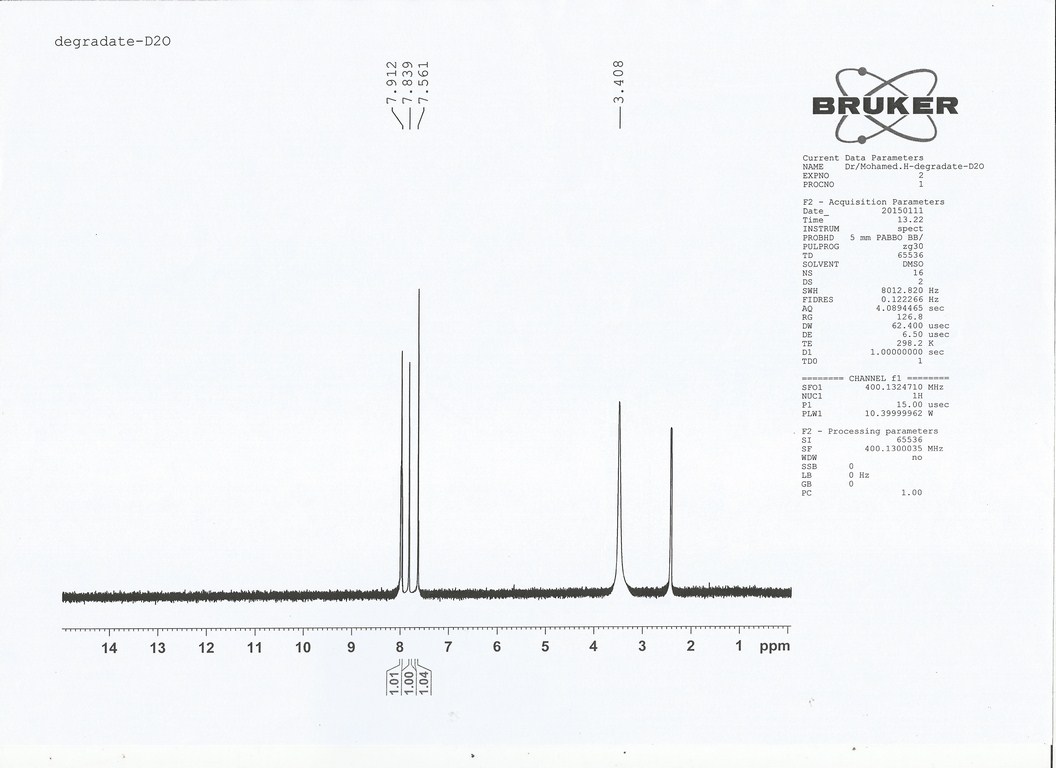
**

**Figure S6. ^1^H NMR spectrum of desmopressin acetate (DPA) Acid-degradants in deuterated (DMSO).**


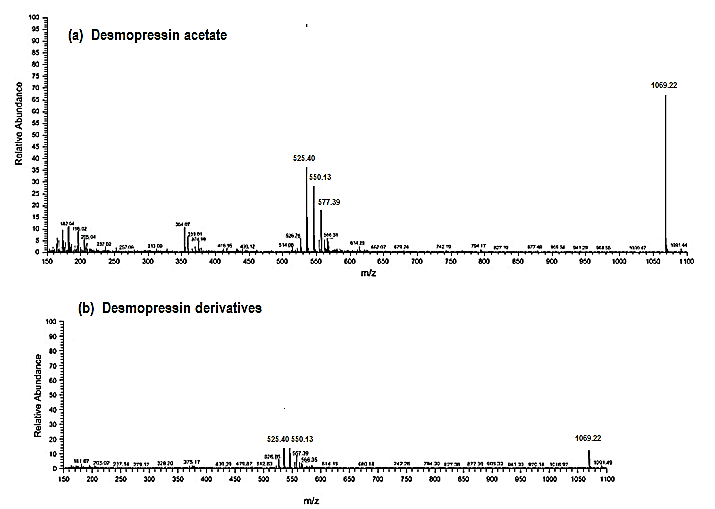


**Figure S7. Mass spectrums of desmopressin acetate (DPA) degradants.**


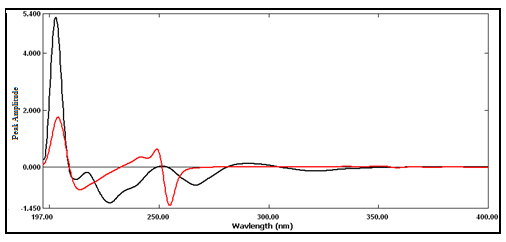


**Figure S8. First-derivative spectra of intact desmopressin acetate (DPA) (―) and its degradation products (——) in methanol.**


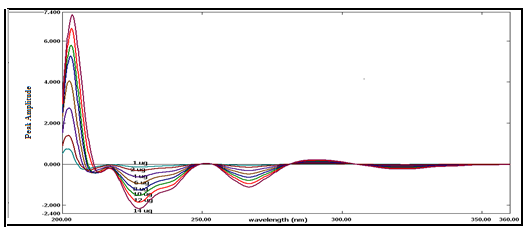


**Figure S9. First derivative of the absorption spectra of desmopressin acetate**

**(DPA) at various concentrations (1-14 µg/mL).**


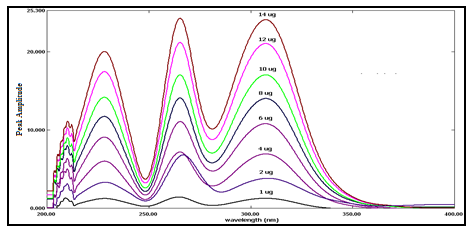


**Figure S10. Ratio spectra of desmopressin acetate (DPA) (1 -14 µg/mL)**

**using (6 µg/mL) DPA acid-degradants as divisor and methanol as blank.**

**
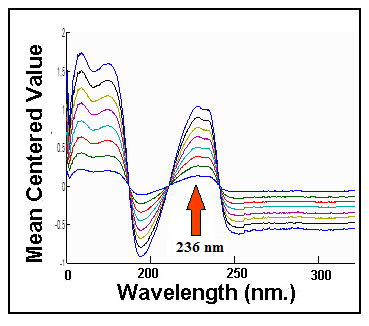
**

**Figure S11.** **Mean centered ratio spectra of desmopressin acetate (DPA)**

**(1 – 14 µg/mL) using (6 µg/mL) of its degradants as a divisor and methanol as blank.**

| **Method** | Conc.  (µg/mL) | **Intraday** | | **Interday** | |
| --- | --- | --- | --- | --- | --- |
|  |  | **Accuracy**  **(R%) ±SD** | **Precision**  **(RSD %)** | **Accuracy**  **(R%)±SD** | **Precision**  **(RSD %)** |
| **First derivative** | 2 | 99.93 ± 1.391 | 1.392 | 100.41 ± 0.817 | 0.814 |
|  | 6 | 99.51 ± 1.227 | 1.233 | 101.34 ± 0.613 | 0.605 |
|  | 12 | 100.54 ± 0.767 | 0.763 | 99.76 ± 1.533 | 1.537 |
| **Ratio derivative** | 2 | 100.53 ± 0.728 | 0.724 | 99.05 ± 0.679 | 0.685 |
|  | 6 | 100.21 ± 0.934 | 0.932 | 98.63 ± 0.570 | 0.578 |
|  | 12 | 99.86 ± 0.252 | 0.253 | 100.19 ± 0.746 | 0.744 |
| **Ratio difference** | 2 | 100.66 ± 0.406 | 0.403 | 100.83 ± 0.233 | 0.231 |
|  | 6 | 99.67 ± 0.646 | 0.648 | 99.91 ± 0.104 | 0.105 |
|  | 12 | 100.95 ± 0.187 | 0.185 | 99.79 ± 0.713 | 0.714 |
| **Mean centering** | 2 | \| 101.59 ± 0.481 \| \| --- \| \|  \| | 0.473 | 99.64 ± 1.285 | 1.290 |
|  | 6 | \| 100.22 ± 0.640 \| \| --- \| \|  \| | 0.639 | 98.94 ± 0.637 | 0.644 |
|  | 12 | 99.12 ± 1.158 | 1.168 | 99.49 ± 0.971 | 0.976 |
| **Dual wavelength** | 2 | 100.45 ± 0.572 | 0.569 | 99.79 ± 0.476 | 0.477 |
|  | 6 | 99.09 ± 0.952 | 0.961 | 99.29 ± 0.951 | 0.958 |
|  | 12 | 100.60 ± 0.579 | 0.575 | 101.44 ± 0.746 | 0.735 |

**Table S1. The intraday and interday precision of the proposed methods**

| **Taken (µg/ml)** | **Added standard (µg/mL)** | | **Recovery % of standard** | | | | |
| --- | --- | --- | --- | --- | --- | --- | --- |
|  |  |  | First derivative | Ratio derivative | Ratio difference | Mean centering | Dual wavelength |
| **4** | | **2** | 98.17 | 99.45 | 101.54 | 99.07 | 98.69 |
|  |  | **4** | 100.45 | 99.03 | 99.17 | 98.13 | 101.08 |
|  |  | **6** | 99.61 | 98.24 | 100.32 | 98.24 | 99.47 |
|  |  | **10** | 101.20 | 99.17 | 99.83 | 99.38 | 100.62 |
| **Mean** | | | 99.86 | 98.97 | 100.22 | 98.71 | 99.97 |
| **RSD%** | | | 1.301 | 0.524 | 0.999 | 0.623 | 1.087 |

**Table S2. Application of standard addition technique to the analysis of Omegapress^®^ tablets using the proposed methods**
